# Impact of stress resilience and susceptibility on fear learning, anxiety, and alcohol intake

**DOI:** 10.1016/j.ynstr.2021.100335

**Published:** 2021-05-06

**Authors:** Sarah T. Gonzalez, Vincent Marty, Igor Spigelman, Steven P. Reise, Michael S. Fanselow

**Affiliations:** a Department of Psychology, University of California, Los Angeles, CA, USA; b Staglin Center for Brain and Behavioral Health, University of California, Los Angeles, CA, USA; c Division of Oral Biology & Medicine, School of Dentistry, University of California, Los Angeles, CA, USA; d Brain Research Institute, University of California, Los Angeles, CA, USA; e Department of Psychiatry and Biobehavioral Sciences, University of California, Los Angeles, CA, USA

**Keywords:** Stress, Fear, PTSD, Resilience, Alcohol use disorder

## Abstract

Post-traumatic stress disorder (PTSD) can develop after exposure to traumatic events and severely impacts the quality of life. PTSD is frequently comorbid with substance use disorders, with alcoholism being particularly common. However, not everyone who experiences trauma develops PTSD and the factors that render individuals susceptible or resilient to the effects of stress are unknown although gender appears to play an important role. Rodent models of stress exposure such as stress-enhanced fear learning (SEFL) recapitulate some aspects of PTSD symptomology, making them an invaluable tool for studying this disorder. This study examined whether exposure to a modified version of the SEFL procedure (4 footshocks instead of the standard 15 over 90 min) would reveal both susceptible and resilient subjects. Following stress exposure, distinct susceptible and resilient groups emerged that differed in fear learning and anxiety-related behavior as well as voluntary alcohol intake. Some aspects of stress susceptibility manifested differently in males compared to females, with susceptibility associated with increased alcohol intake in males and increased baseline anxiety in females.

## 1 Introduction

Post-traumatic stress disorder (PTSD) may develop after exposure to traumatic events and severely impacts the quality of life. PTSD patients experience a number of debilitating symptoms including hypervigilance and avoidance of stimuli reminiscent of the trauma (APA, 2013). PTSD is also often co-morbid with drug abuse, with alcohol abuse being the most common (Kessler et al., 1995; Mills et al., 2006; Perkonigg et al., 2000). Despite the high burden of this disorder, its mechanisms are not fully understood and currently available treatments such as exposure therapy are not always effective (Craske et al., 2008; Hembree et al., 2003; Milad et al., 2009).

A critical question is why some individuals develop the disorder following trauma while others do not. It has been estimated that while approximately one third of the population will experience a trauma during their lifetime, only 10–20% of these individuals will develop PTSD with women twice as likely to develop the disorder compared to men (Brunello et al., 2001; Kessler et al., 1995, 2017). Furthermore, not all PTSD patients display the same types or intensity of symptoms, and neuroimaging studies suggest that different categories of symptoms may be supported by different neural mechanisms (Lanius et al., 2001, 2002, 2006). Determining the factors that promote susceptibility or resilience to developing the disorder is therefore crucial for understanding the disorder and developing more effective, targeted treatments.

Animal models that capture some of the behavioral symptoms of PTSD are a powerful tool to study the mechanisms of this disorder. One characteristic of PTSD is an exaggerated response to mild stressors that are reminiscent of the original trauma, leading to inappropriate fear responses (Bremner et al., 1995; Dykman et al., 1997). Our laboratory has developed a rodent model of stress exposure termed stress-enhanced fear learning (SEFL) that uses aspects of Pavlovian fear conditioning to capture this exaggerated fear response. In standard Pavlovian fear conditioning, a neutral conditional stimulus (CS), such as a discrete cue or a context, is paired with an aversive unconditional stimulus (US), such as a footshock. Following the formation of a CS-US association, the CS will elicit a fear response. In the SEFL model, animals are exposed to an intense acute stressor (typically 15 unsignaled footshocks) in a distinct context, followed by a mild fear conditioning event (a single footshock) in a different conditioning context (Rau et al., 2005). Following stress exposure animals show exaggerated fear to the fear conditioning context. This enhancement of fear learning is the defining feature of the SEFL model and reflects a nonassociative sensitization of the fear learning process as it is not prevented by amnesia for, or extinction of, fear to the stress context (Long and Fanselow, 2012; Poulos et al., 2014; Rau et al., 2005). Additionally, the acute stress will also enhance future fear learning to an auditory stimulus paired with shock even though there are no auditory stimuli present during stress (Pennington et al., 2017).

While enhanced fear learning is the key feature of the SEFL model, this procedure produces a number of other behavioral changes reflective of PTSD symptoms including extreme fear of stimuli associated with the original stressor, increased anxiety-related behavior, increased startle reactivity, and altered glucocorticoid signaling (Meyer et al., 2013; Pennington et al., 2017; Perusini et al., 2016; Poulos et al., 2015). Importantly, the SEFL model produces a long-lasting increase in voluntary alcohol intake, reflecting the comorbidity between PTSD and substance abuse (Meyer et al., 2013).

While a number of stress exposure models have been used to study PTSD, these models often consist of unquantifiable stressors whose parameters cannot be quantitatively manipulated such as social defeat stress, predator odor, or combinations of multiple stressors (Cohen et al., 2006; Golden et al., 2011; Willner et al., 1987). In addition, few studies have examined the responses of males and females to the same stressor despite the fact that women are twice as likely to develop PTSD compared to men after adjusting for stress severity (Goldstein et al., 2016; Kessler et al., 1995). The stressor typically used in our experiments (15 unsignaled footshocks over 90 min) produces a robust enhancement in fear learning in both males and females that is advantageous for studying the mechanisms of this enhancement but may obscure individual differences that promote susceptibility versus resilience to the effects of stress. However, this stressor is uniquely quantifiable such that stress severity can be readily manipulated by changing the number or amplitude of footshocks. By reducing the stressor severity to 4 footshocks, we sought to uncover both susceptible and resilient groups and determine whether females show increased stress sensitivity.

Many studies investigating stress susceptibility rely on post-hoc methods such as quantifying the behavioral outcome of interest in the data set under study (e.g. increased freezing or reduced social interaction) and defining the 20–30% of subjects at one extreme as susceptible and the 20–30% at the other extreme as resilient (Colucci et al., 2020; Covington et al., 2010; Jeong et al., 2020; Ritov et al., 2016). These methods of classification are limited as they are based just on the current sample and similar procedures are rarely applied to unstressed controls. We aimed to improve upon existing methods of classification by establishing an *a priori* criterion based on prior sampling of a large population of subjects showing a normal fear response.

In the following experiments we tested the hypothesis that using a novel *a priori* classification criterion would reveal both susceptible and resilient populations following a reduced 4-footshock stressor. We further hypothesized that susceptible subjects would demonstrate a constellation of behavioral differences relevant to PTSD symptomology. We found that using a reduced-severity stressor produced distinct susceptible and resilient groups that differed on several measures of fear, anxiety, and alcohol-related behaviors. We further found that while males and females did not differ in rates of stress susceptibility, susceptible males and females showed different patterns of alcohol consumption and anxiety-related behavior.

## 2 Methods

### 2.1 Animals

66 adult male and female Long-Evans rats (Envigo) were used, approximately 75 days old at the start of the experiment. Animals were individually housed under a 12-h light/dark cycle. Food and water were available ad lib in the home cage, and during intermittent access two-bottle choice enrichment in the form of paper twists was provided. Animals were handled daily for 60 s each day for 1 week prior to the start of the experiment. The UCLA Institutional Animal Care and Use Committee approved all procedures involving animals.

### 2.2 Establishment of “susceptible” versus “resilient” classification criterion

In the standard SEFL procedure, rats are first exposed to a stressor consisting of 15 unsignaled 1-sec, 1-mA footshocks or receive equivalent context exposure without footshock. Subjects then receive a mild fear conditioning event consisting of a single footshock in a novel context. During the critical test of the SEFL procedure, termed the SEFL test, subjects are returned to the fear conditioning context for 8 min during which fear is assessed via freezing. The key feature of SEFL is that stress-exposed subjects show enhanced fear to the fear conditioning context relative to unstressed controls.

To determine an *a priori* criterion for classifying whether performance on the SEFL test deviated from that of unstressed controls, we compiled SEFL test scores from a large number of unstressed control subjects (n = 182), drawing from published and unpublished SEFL experiments previously run in our laboratory. These data were pooled over several years, but all experiments used male and/or female young adult Long-Evans rats (approximately 9–12 weeks old) and the same training parameters including number and intensity of footshock.

Subjects were classified as “Susceptible” if they showed performance on the SEFL test that was at least two standard deviations above the means of unstressed controls, which based on this dataset, was 58% freezing (Mean = 18.8%, SD = 19.6%). Subjects that showed less than 58% freezing during the SEFL test were classified as “Resilient”.

**Fig. 1:**
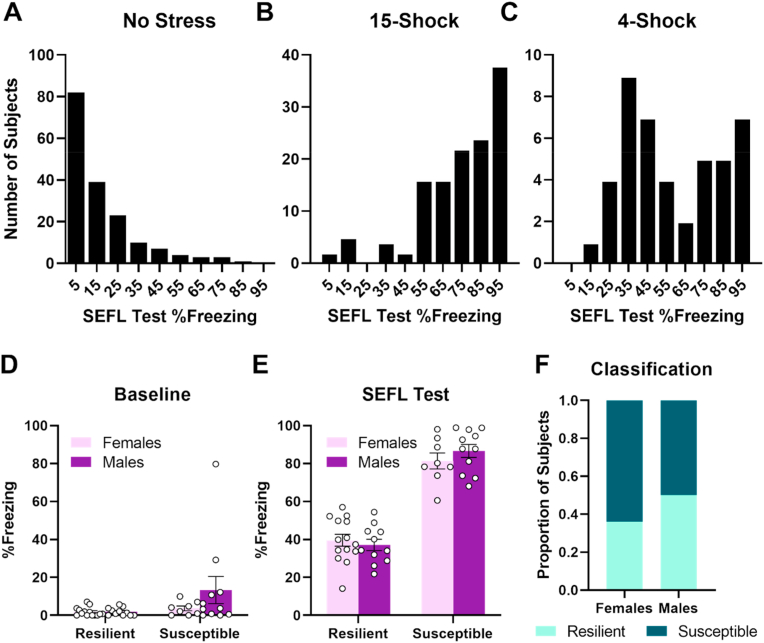
Stress-enhanced fear learning following different levels of stress. **A.** Distribution of SEFL test scores in unstressed controls (n = 182 drawn from previous published and unpublished experiments). Tick marks indicate center of 10% bins. **B.** Distribution of SEFL test scores in subjects exposed to standard 15-footshock stress (n = 130 drawn from previous published and unpublished experiments). **C.** Distribution of SEFL test scores in subjects exposed to 4-footshock stress in the present experiment (n = 44). **D.** Susceptible and Resilient subjects do not differ in baseline fear during the 3 min prior to footshock delivery (Day 18). **E.** No differences between males and females were observed during the SEFL test (Day 19). **F.** Proportion of subjects classified as Susceptible or Resilient. Error bars represent standard error of the mean.

Nearly all unstressed control subjects (94.0%) met the Resilient criterion (distribution of SEFL test scores shown in Fig. 1A). Conversely, the same analysis performed on subjects exposed to the standard 15-shock stress (n = 130) revealed that the majority of stressed subjects (79.2%) met the Susceptible criterion (Mean = 74.5%, SD = 22%, distribution of SEFL test scores shown in Fig. 1B). This classification criterion was therefore used to determine whether the 4-footshock stress would produce both Susceptible and Resilient populations (experimental timeline shown in Table 1).

**Table 1:** Experimental timeline.

Day	Task
1–14	Continuous access 2-bottle choice
15	Light-dark transition test
16	Stress
17	Generalization test
18	Single footshock
19	Stress-enhanced fear learning test
20	Open field test
21	Elevated plus maze
22	Light-dark transition test
23–27	Extinction
28	Aversive acoustic stimulus
29	Context test
30-37 Rest	
38–97	Intermittent access 2-bottle choice

Given that PTSD is more common in women than in men, we evaluated whether the susceptibility cutoff would differ if calculated separately for males and females (Supplementary Methods). We found no differences in the distribution of SEFL test scores between unstressed males and females (Figs. S1A–B), or in males and females exposed to the standard 15-footshock stress (Figs. S1C–D). Importantly, we found that the susceptibility criterion remained the same when calculated separately for males and females (Supplementary Methods). It has been reported that female rats express fear via darting behavior, but to our knowledge this has only been reported during auditory fear conditioning (Gruene et al., 2015). Videos were screened for darting, but we did not observe any darting behavior in either males or females during the SEFL test.

Our approach focused on determining a criterion for susceptibility, i.e. identifying the population of subjects that show unusually high levels of fear. An alternative approach would be to determine the criterion for resilience, i.e. identifying stress-exposed subjects that show unusually low levels of fear. We performed a similar analysis to consider this alternative that was based on the 15 footshock-exposed subjects, but determined that this approach did not perform as well (Supplementary Methods).

### 2.3 Assessments of baseline alcohol consumption and anxiety-related behavior

#### 2.3.1 Continuous access two-bottle choice (2BC) drinking procedure

All fluids were presented in 250-ml drinking bottles with low-leak drinking spouts (Ancare) accessible through the top of the home cage. Rats had access to one bottle containing 10% EtOH (w/v) and one bottle containing regular drinking water. Rats received continuous access to both bottles for two weeks, which in contrast to intermittent access produces relatively low levels of alcohol consumption (Brancato et al., 2016; Wise, 1973). Bottles were weighed daily except Sundays and locations were alternated daily to control for side preferences. Rats were weighed every other day to calculate the grams of solution consumed per kilogram of body weight in each 24-h session (g/kg/24 h).

#### 2.3.2 Light-dark transition test

The light-dark transition test is a classic test of anxiety-related behavior in rodents (Bourin and Hascoët, 2003; Crawley and Goodwin, 1980). The apparatus consisted of a Plexiglas arena (100 cm × 40 cm x 30 cm). The arena was divided into a light compartment with white walls and floors (75 cm × 40 cm x 30 cm) and a dark compartment with black walls and floors (25 cm × 40 cm x 30 cm), with a 10 cm × 10 cm opening allowing movement between the two compartments. The room was dimly lit by a lamp in the corner of the room. Each session was recorded using an overhead camera and behavior automatically tracked using EthoVision software (Noldus). Subjects were individually placed in the dark compartment of the light-dark transition test apparatus and allowed to freely move between the dark and light compartments for 10 min. Anxiety-related behavior was scored by the latency to enter the light compartment, the total time spent in the light compartment and the number of entries into the light compartment.

Unlike many rodent tests of anxiety-related behavior, previous reports indicate that the light-dark transition test can be reliably conducted multiple times within the same subject (Ballaz et al., 2007; Banasikowski et al., 2015; Onaivi and Martin, 1989). The light-dark transition test was therefore run both prior to and following stress exposure while all other assessments of anxiety-related behavior were run only following stress exposure.

### 2.4 Fear conditioning

#### 2.4.1 Stress-enhanced fear learning

All fear conditioning took place in four sets of four identical fear conditioning chambers housed in sound-attenuating shells (Med-Associates). Context A (stress context) contained flat grid floors, was lit by a white house light and scented with 50% Windex solution. Ventilation fans provided background noise. Context B (generalization context) was identical to Context A except the grid floors were made of alternating thick and thin bars and chambers were scented with 1:30 Simple Green solution. Context C (mild fear conditioning context) contained a black triangular insert and floors composed of grid bars at alternating vertical heights and was scented with 1% acetic acid. Each set of grids was wired to a shock generator and scrambler. Sessions were recorded by near-infrared cameras and freezing was automatically scored using VideoFreeze software (Med Associates).

Subjects were first transported to Context A in their homecages where they received an acute stressor consisting of 4 1-sec, 1-mA unsignalled footshocks randomly distributed over 90 min. One day later, fear generalization was assessed by exposing subjects to a novel context that shared some features with the original stress context (Context B) for 8 min without footshock. The next day subjects were transported to Context C in a black plastic tub divided into 4 quadrants. Following a 3-min baseline period, all subjects received a single 1-sec, 1-mA footshock and were removed 30 s later. All subjects were returned to Context C the following day for 8 min to assess fear to the mild fear conditioning context. Freezing served as our index of fear conditioning.

#### 2.4.2 Fear extinction

To assess fear extinction to the mild fear conditioning context (Context C), subjects received 5 days of extinction training, in which they were exposed to Context C for 30 min per day without footshock delivery. Extinction across sessions was measured by freezing during the first 5 min of each session. To account for differences in initial fear levels, extinction was also measured by the number of sessions required for each subject to reach 50% of their freezing on the first day of extinction. Subjects that did not reach this criterion were given a score of 5.

#### 2.4.3 Aversive acoustic stimulus

Context D (white noise context) contained a curved white plastic wall and white plastic floor inserts and was scented with 1:30 Simple Green solution. Acoustic stimuli were delivered using Goldwood GT-1005 wide dispersion piezo tweeters mounted to the wall of the chambers and connected to an amplifier. Following a 3-min baseline period, all subjects received a 100-msec, 110-dB burst of white noise and were removed 30 s later. The next day subjects were returned to Context D for 8 min to assess fear to the context.

### 2.5 Assessments of anxiety-related behavior following stress

#### 2.5.1 Open field test

This task utilized a modified version of the open field test, a classic test of anxiety-related behavior in rodents (Godsil et al., 2005; Godsil and Fanselow, 2004). The apparatus consisted of a clear plastic container (78 cm × 39 cm x 30 cm) divided into 4 equal-sized zones (19.5 cm × 39 cm). Three LED lamps were located around each end of the apparatus, one lamp located against the center of each short wall and one lamp located against the ends of each long wall. Turning on the lamps on one end created a light gradient across the apparatus, with Zone 1 the brightest (2160 Lux) and Zone 4 the dimmest (260 Lux). The illuminated side of the apparatus was counterbalanced across subjects. The room was lit by a red light throughout the session, and each session was recorded using an overhead camera and behavior automatically tracked using EthoVision software.

Subjects were individually placed in the open field apparatus for 12 min and allowed to roam freely. During the first 4 min, the apparatus was lit only by a red light. During minutes 5–8, the lamps around one side of the apparatus were illuminated creating a light gradient across the floor. During minutes 9–12, the lamps were turned off. To assess anxiety-related behavior, general exploratory behavior was measured throughout the session and by the amount of time spent in Zone 4, indicating avoidance of the lights.

#### 2.5.2 Elevated plus maze

The elevated plus maze is another classic rodent test of anxiety-related behavior (Rodgers and Dalvi, 1997). The apparatus consisted of a plus-shaped maze with each arm measuring 40 cm × 12.5 cm that was elevated 60 cm above the ground. The closed arms had black Plexiglas walls (17.5 cm), while the open arms did not. The room was lit by a red light throughout the session, and each session was recorded using an overhead camera and behavior automatically tracked using EthoVision software.

Subjects were placed in the center of the maze and allowed to freely explore for 6 min. Anxiety-related behavior was measured by the amount of time spent in the open arms of the maze, with decreased time indicating increased anxiety. 14 subjects (6 females and 8 males) fell off of the maze and were excluded from analysis of this task.

### 2.6 Intermittent access two-bottle choice (2BC) drinking paradigm

Subjects received 59 days of intermittent access 2-bottle choice as described previously (Meyer et al., 2013), which has been shown to produce escalating levels of alcohol consumption (Simms et al., 2008; Wise, 1973). During this time subjects were given access to one bottle of 10% ethanol (EtOH, w/v) and one bottle of regular drinking water on Mondays, Wednesdays and Fridays. Drinking bottles were weighed at the beginning and end of each 24-h drinking session and rats were weighed at the end of each session. Bottle locations were alternated each session. At the end of each session the bottle containing alcohol was replaced with a bottle containing drinking water until the start of the next session.

### 2.7 Data analysis

To evaluate whether Susceptible and Resilient subjects differed on fear, anxiety and alcohol consumption-related behavior, performance on each individual measure was analyzed using IBM SPSS Statistics Software. Tasks with a single dependent variable were analyzed with 2-way ANOVA with Resilience as the first factor and Sex as the second factor. Tasks with repeated measures were analyzed using mixed-model ANOVA. Significant interactions were interpreted using simple main effects. Violations of sphericity in mixed-model ANOVA were addressed by adjusting the degrees of freedom (dfs) using the Greenhouse-Geisser correction.

Analysis of whether baseline anxiety levels predicted post-stress task performance was performed using R 3.6.3 (R Core Team, 2020), the mosaic package (Pruim et al., 2017) and the psych package (Revelle, 2019). Principal component analysis was first performed to compute a single measure of baseline anxiety from the 3 measures of anxiety-related behavior from the light-dark transition test (entries, light time, latency). Backwards regression was then performed to predict each post-stress measure from the principal component analysis variable, sex and the interaction term resampling 1000 times for each test. If the interaction term did not explain a significant proportion of variance it was removed from the analysis. Principal component analysis was also performed to compute a single measure of anxiety from the second light-dark transition test conducted following stress.

## 3 Results

### 3.1 4-Shock stress produces distinct susceptible and resilient populations

Applying our *a priori* classification criterion to the results from previous studies indicated that unstressed control subjects are predominantly classified as Resilient, while subjects exposed to the standard 15-footshock stress are mainly classified as Susceptible (Fig. 1A–B). In contrast, the presently used 4-shock stress produced a pronounced bimodal distribution, with 56.8% of subjects classified as Resilient and 43.2% classified as Susceptible (Fig. 1C).

To verify that the differences between these groups during the SEFL test reflected differences in fear learning, rather than differences in generalization of fear from the stress context, we examined freezing in the fear conditioning context prior to footshock delivery. No differences in baseline fear were observed between Susceptible and Resilient subjects (Resilience: F_1,40_ = 3.18, p = 0.08; Sex: F_1,40_ = 1.75, p = 0.19; Resilience*Sex: F_1,40_ = 1.69, p = 0.20; Fig. 1D).

We next examined whether females were more likely to be classified as Susceptible compared to males. SEFL test performance did not differ between males and females (Sex: F_1,40_ = 0.17, p = 0.69; Sex*Resilience: F_1,40_ = 1.24, p = 0.27; Fig 1E; Figs. S1E–F). While slightly fewer females were classified as Susceptible compared to males (Females: 36%; Males: 50%; Fig. 1F), a chi-square test revealed no sex differences in the number of subjects classified as Susceptible (X^2^(1,N = 44) = 0.83, p = 0.36). These results indicate that the 4-footshock stress can be used to reveal distinct Susceptible and Resilient populations, although males and females do not appear to differ in stress susceptibility. Performance of Susceptible and Resilient subjects on the battery of tasks used in this experiment compared to unstressed controls is shown in Table S1 (Supplementary Materials).

### 3.2 Susceptible subjects show increased fear generalization following stress exposure

**Fig. 2:**
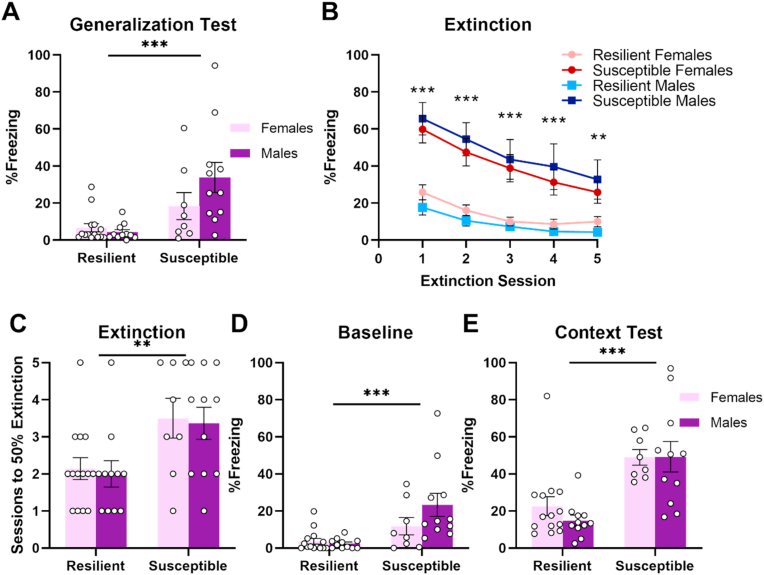
Susceptible and Resilient subjects differ on multiple aspects of fear learning. **A.** Susceptible subjects show increased fear generalization during the generalization test (Day 17). **B.** Susceptible subjects show increased fear throughout fear extinction (Days 23–27). Data points show freezing during the first 5 min of each session. **C.** Susceptible subjects require more sessions to reach 50% of the initial freezing levels on the first day of extinction. **D.** Susceptible subjects show increased baseline fear during the 3 min prior to acoustic stimulus delivery (Day 28). **E.** Susceptible subjects show increased fear to acoustic stimulus context (Day 29). **p < 0.01, ***p < 0.001. Error bars represent standard error of the mean.

To fully characterize the differences between Susceptible and Resilient subjects we first assessed whether these subjects differed on other aspects of fear learning that are relevant to PTSD symptomology. Following stress exposure but prior to receiving the single footshock, fear generalization was assessed by exposing subjects to a novel context that shared some features with the original stress context. Susceptible subjects showed increased fear generalization compared to Resilient subjects, (F_1,40_ = 15.74, p < 0.001; Fig. 2A), although there were no differences between males and females (Sex: F_1,40_ = 1.60, p = 0.21; Sex*Resilience: F_1,40_ = 2.96, p = 0.09).

During fear extinction to the fear conditioning context, significant differences between Susceptible and Resilient subjects were observed (Session*Resilience: F_2.06,82.41_ = 4.64, p = 0.012, dfs adjusted using Greenhouse-Geisser correction; Fig. 2B), with Susceptible subjects showing elevated freezing during all sessions (ps < 0.01–0.001). To compare extinction rates between groups in a manner to compensate for the initial differences in freezing levels, we measured the number of extinction sessions required to reach 50% of initial freezing levels displayed on the first day of extinction. Results indicated that Susceptible subjects may show impaired fear extinction as they required a greater number of sessions to reach this criterion (F_1,40_ = 11.82, p = 0.001; Fig. 2C). Males and females did not differ during extinction (Sex: F_1,40_ = 0.02, p = 0.90; Resilience*Sex: F_1,40_ = 1.13, p = 0.29) or in the number of sessions required to reach criterion (Sex: F_1,40_ = 0.12, p = 0.73; Sex*Resilience: F_1,40_ = 0.001, p = 0.99).

### 3.3 Susceptible subjects show increased fear to an aversive acoustic stimulus

While Susceptible subjects showed increased fear to a stimulus reminiscent of the original stress, it was unknown whether these subjects would show similarly enhanced fear to a novel aversive stimulus. Subjects were placed in a novel context (Context D) in which they received an aversive acoustic stimulus consisting of a 110-dB, 100-ms burst of white noise. Susceptible subjects showed greater fear to the white noise context (F_1,40_ = 27.77, p < 0.001; Fig. 2E), although no differences between males and females were observed (Sex: F_1,40_ = 0.41, p = 0.53; Sex*Resilience: F_1,40_ = 0.48, p = 0.49). However, Susceptible subjects also showed increased freezing prior to stimulus delivery (F_1,40_ = 14.79, p < 0.001; Fig. 2D), suggesting that the elevated fear seen during test could potentially be due to generalization of fear from a previously experienced context.

To address this possibility, a separate group of rats received both the 4-shock stress and mild fear conditioning but underwent extinction training to Context D prior to white noise delivery to eliminate any differences in baseline fear (Supplementary Methods; Figs. S2A–E). While Susceptible subjects initially showed greater fear to the white noise context (F_1,41_ = 6.02, p = 0.02, Fig. S2E), by the final extinction session there was no difference between Susceptible and Resilient subjects (F_1,41_ = 2.22, p = 0.14). While Susceptible and Resilient subjects did not differ in fear during the 3 min prior to stimulus onset (F_1,41_ = 0.31, p = 0.58; Fig. S2F), Susceptible subjects showed significantly elevated fear to the context the following day (F_1,41_ = 5.12, p = 0.03; Fig. S2G). While this effect appeared stronger in the females, the interaction did not reach significance (F_1,41_ = 2.45, p = 0.13). These results indicate that Susceptible subjects show increased fear to a context paired with a novel aversive stimulus in addition to a stimulus reminiscent of the original stress.

### 3.4 Susceptible subjects show increased anxiety-related behavior following stress

**Fig. 3:**
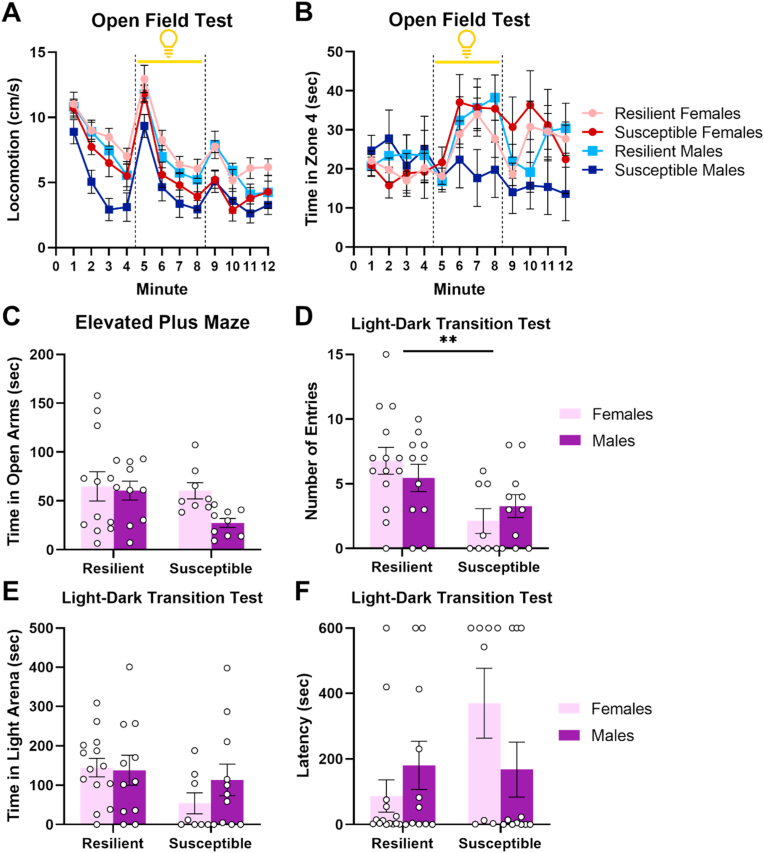
Susceptible subjects show increased anxiety-like behavior following stress exposure. **A.** Susceptible subjects show decreased locomotion during the open field test (Day 20). Dotted lines indicate transitions from lights off to on (first line) and lights on to off (second line). **B.** Susceptible males show a blunted response to light onset in comparison to Resilient males. **C.** No differences in time spent in the open arms of the elevated plus maze (Day 21). **D-F.** Results of the light-dark transition test (Day 22). Susceptible subjects show reduced entries into the light compartment (**D**), marginally decreased time in the light compartment (**E**) and marginally increased latency to enter the light compartment (**F**). **p < 0.01. Error bars represent standard error of the mean.

We have previously shown that stress exposure increases anxiety-related behavior (Perusini et al., 2016). We therefore examined whether Susceptible subjects showed heightened anxiety using a battery of behavioral tasks following stress exposure. Susceptible subjects showed increased anxiety-related behavior as indicated by reduced exploratory behavior during the open field test (F_1,40_ = 12.43, p = 0.001; Fig. 3A) and decreased entries into the light compartment during the light-dark transition test (F_1,40_ = 10.86, p = 0.002; Fig. 3D). Numerical differences were also observed in time spent in the light compartment (F_1,40_ = 2.94, p = 0.09; Fig. 3E), and latency to enter the light compartment (F_1,40_ = 3.16, p = 0.08; Fig. 3F), but these effects fell short of statistical significance.

Analysis of time spent in Zone 4 of the open field test revealed a strong trend towards an interaction between time, sex, and stress susceptibility (F_5.88,235.15_ = 1.97, p = 0.07, dfs adjusted using Greenhouse-Geisser correction; Fig. 3B). Female subjects showed light avoidance with no observed effects of Resilience (F_1,20_ = 0.27, p = 0.61) or Resilience by Minute interaction (F_4.79,95.78_ = 0.83, p = 0.53, dfs adjusted using Greenhouse-Geisser correction). In contrast, Susceptible males showed reduced time spent in Zone 4 during and after light onset as indicated by a significant Resilience by Minute interaction (F_5.02,100.38_ = 2.43, p = 0.04, dfs adjusted using Greenhouse-Geisser correction). These results suggest that stress susceptibility is uniquely associated with a blunted response to light onset in males.

Time spent in the open arms of the elevated plus maze was also analyzed as a measure of anxiety-related behavior. However, no effects of sex or resilience were observed (Resilience: F_1,35_ = 2.71, p = 0.11; Sex: F_1,35_ = 2.66, p = 0.11; Sex*Resilience: F_1,35_ = 1.57, p = 0.22; Fig. 3C). Lastly, we took advantage of the fact that the light-dark transition test had been used prior to and following stress to test the possibility that stress exposure differentially altered performance in Susceptible versus Resilient subjects. However, no interactions between stress exposure and resilience were observed on number of entries into the light arena (F_1,40_ = 1.69, p = 0.2; Fig. S3A), time in the light arena (F_1,40_ = 0.07, p = 0.8; Fig. S3B) or latency to enter the light arena (F_1,40_ = 1.58, p = 0.22; Fig. S3C).

### 3.5 Susceptible subjects show increased alcohol consumption in the absence of pre-stress alcohol exposure

**Fig. 4:**
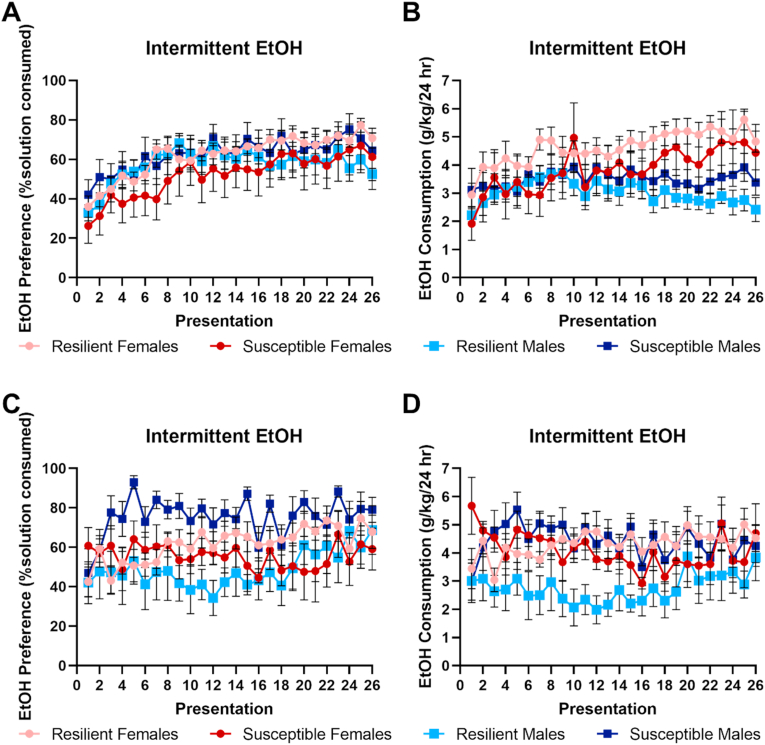
Results of intermittent access 2-bottle choice following stress exposure (Days 38-97). **A-B.** Results of subjects exposed to alcohol prior to stress exposure. No differences were observed in alcohol preference **(A)**, although females showed increased consumption compared to males **(B)**. **C-D.** Results of subjects not exposed to alcohol prior to stress exposure. In comparison to Resilient males, Susceptible males showed increased alcohol preference **(C)** and alcohol consumption **(D)**. Error bars represent standard error of the mean.

Susceptibility did not appear to alter alcohol consumption following stress in subjects that had also been exposed to alcohol prior to stress. Differences between males and females emerged across presentations in alcohol consumption, with females showing increased consumption (F_7.68,307.19_ = 3.76, p < 0.001, dfs adjusted using Greenhouse-Geisser correction; Fig. 4B). However, no difference in alcohol preference was observed (F_7.61,304.57_ = 1.43, p = 0.19, dfs adjusted using Greenhouse-Geisser correction; Fig. 4A). There were no differences between Susceptible and Resilient subjects in either alcohol consumption (Resilience: F_1,40_ = 0.12, p = 0.73; Sex*Resilience: F_1,40_ = 1.88, p = 0.18) or alcohol preference (Resilience: F_1,40_ = 0.17, p = 0.69; Sex*Resilience: F_1,40_ = 1.44, p = 0.24).

Previous work indicates that while stress increases alcohol consumption, this effect is blocked if rats had established drinking habits via intermittent EtOH exposure prior to stress (Meyer et al., 2013). This suggests that alcohol access prior to stress exposure may have blunted potential differences between Susceptible and Resilient subjects. To address this possibility, a separate group of subjects received identical treatment except both bottles during continuous alcohol access prior to stress contained only drinking water.

A significant interaction between sex and resilience was observed in alcohol preference following stress exposure (F_1,18_ = 7.18, p = 0.02; Fig. 4C). Susceptible males showed increased alcohol preference compared to Resilient males (F_1,18_ = 10.20, p = 0.005), while no differences were observed in females (F_1,18_ = 0.41, p = 0.53). A similar trend was seen in alcohol consumption (F_1,18_ = 3.69, p = 0.07; Fig. 4D). Susceptible males showed increased alcohol consumption compared to Resilient males (F_1,18_ = 5.95, p = 0.03) while no difference was observed in females (F_1,18_ = 0.1, p = 0.76). These results indicate that in the absence of prior alcohol exposure, stress susceptibility is associated with increased alcohol intake in males but not in females.

### 3.6 Baseline anxiety is predictive of susceptibility to SEFL in females

**Fig. 5:**
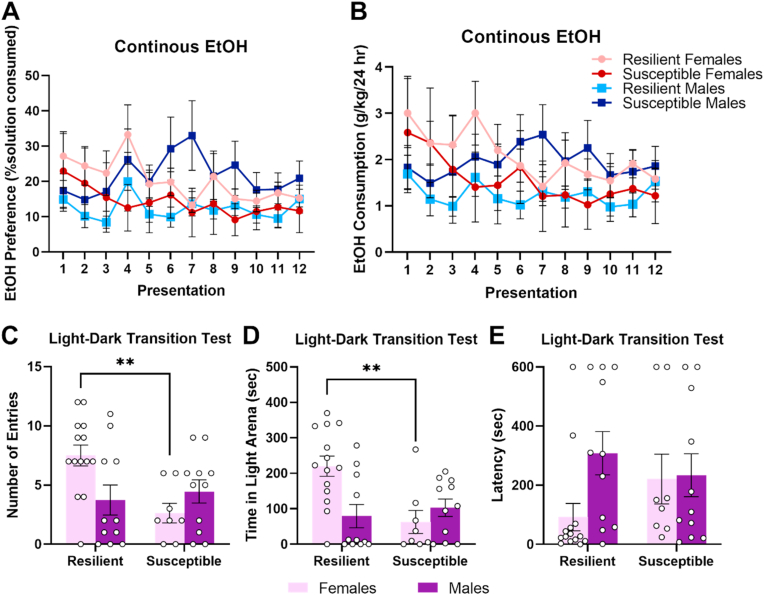
Baseline levels of alcohol consumption and anxiety-like behavior prior to stress exposure. **A-B.** No differences in alcohol preference **(A)** or alcohol consumption **(B)** during continuous access 2-bottle choice (Days 1–14). **C-E.** Susceptible females show increased anxiety on the light-dark transition test (Day 15) compared to Resilient females. Susceptible females showed reduced entries into the light compartment **(C)** and decreased time in the light compartment **(D)**, though no significant difference in latency to enter the light compartment **(E)**. **p < 0.01. Error bars represent standard error of the mean.

Baseline levels of EtOH consumption and anxiety were retrospectively evaluated as potential predictors of susceptibility to stress. EtOH consumption prior to stress did not appear to be a predictor of stress susceptibility as Susceptible and Resilient subjects did not differ on either alcohol preference (F_1,39_ = 0.20, p = 0.66; Fig. 5A) or alcohol consumption (F_1,39_ = 0.07, p = 0.79; Fig. 5B).

In contrast, baseline anxiety was predictive of stress susceptibility in females but not in males. Performance on the light-dark transition test revealed significant interactions between sex and susceptibility on entries into the light compartment (F_1,40_ = 7.21, p = 0.01; Fig. 5C) and time spent in the light compartment (F_1,40_ = 8.77, p = 0.005; Fig. 5D). Follow-up simple main effects revealed that Susceptible females made fewer entries (p = 0.002) and spent less time in the light compartment (p = 0.001) compared to Resilient females. In contrast, Susceptible and Resilient males did not differ on either measure (entries: p = 0.62; light time: p = 0.58). While Susceptible females also showed numerically increased latency to enter the light compartment, this difference was not significant (F_1,40_ = 2.24, p = 0.14; Fig. 5E).

### 3.7 Baseline anxiety predicts several fear and anxiety-related behaviors following stress

Given that baseline anxiety was predictive of subsequent performance on the SEFL test, and that Susceptible subjects showed a constellation of behavioral changes relevant to PTSD symptomology, we next looked at whether baseline anxiety predicted post-stress behaviors.

Baseline anxiety predicted fear learning-related behavior in a sex-specific manner. Backward regression was performed to determine whether baseline anxiety levels predicted key measures of fear learning. Baseline anxiety, sex, and their interaction explained a significant amount of variance in performance on the SEFL test (F_3,40_ = 2.85, p = 0.049, R^2^ = 0.18). The interaction term was found to be significant (β = 18.55, p < 0.05), and follow-up correlations indicated that baseline anxiety in females was correlated with fear during the SEFL test in females (r = −0.59, n = 22, p = 0.004) but not in males (r = 0.15, n = 22, p = 0.51).

This regression model also explained a significant amount of variance in the number of extinction sessions required to reduce fear by 50% (F_3,40_ = 4.29, p = 0.01, R^2^ = 0.24). The interaction term was again significant (β = 1.28, p < 0.01) with lower anxiety associated with slower extinction in males (r = 0.66, n = 22, p = 0.001) while there was no relationship between baseline anxiety and extinction in females (r = −0.20, n = 22, p = 0.38). However, this regression model was insufficient to explain a significant amount of variance in fear to the generalization context (F_3,40_ = 0.98, p = 0.41, R^2^ = 0.07) or the white noise context test (F_3,40_ = 1.91, p = 0.14, R^2^ = 0.13).

In contrast to the effect on fear learning, baseline anxiety generally predicted post-stress anxiety in a sex-independent manner. Baseline anxiety and sex explained a significant amount of variance in average locomotion during the open field test (F_2,41_ = 4.65, p = 0.02, R^2^ = 0.19) and the performance on the light-dark transition test (F_2,41_ = 8.06, p = 0.001, R^2^ = 0.28), though not the elevated plus maze (F_2,36_ = 2.39, p = 0.11, R^2^ = 0.12). Baseline anxiety was found to significantly predict performance during the open field test (β = 0.73, p < 0.05) and the light-dark transition test following stress (β = 0.56, p < 0.001). These findings indicate that despite the large battery of behavioral assays used, we were able to obtain relatively stable assessments of anxiety in each animal.

## 4 Discussion

One of the challenges in understanding and treating PTSD is understanding why some individuals develop the disorder following trauma exposure while others do not. While several rodent models of stress exposure have been developed to study PTSD, a need remains for models that can probe the individual variability in responses to stress in both male and female subjects (Richter-Levin et al., 2019; Shansky, 2015). Here we demonstrate that a modified version of the stress-enhanced fear learning (SEFL) procedure can be used to study the factors that promote susceptibility versus resilience to the effects of stress. Following exposure to an acute stressor consisting of 4 footshocks over 90 min, distinct populations emerge with approximately 60% of subjects classified as Resilient and 40% as Susceptible.

While subjects were classified based on their performance on the SEFL test, these subjects showed alterations in a variety of behaviors relevant to PTSD symptomology, summarized in Table 2. 43% of the rats exceeded our classification criterion following the 4-shock stress, while 79% passed the criterion when 15 shocks were administered. This indicates that the magnitude of the stressful event is one important factor in determining whether PTSD-like symptomatology will develop following trauma. However, given the bimodal split in the 4-shock condition, other pre-existing factors are also important determinants of post-stress reactivity. In females, prestress anxiety is correlated with which subjects will develop these post-stress symptoms. The current procedures and measures may provide a useful tool for probing these individual differences.

**Table 2:** Summary of results indicating distinct Susceptible phenotypes. Results were observed in both males and females unless otherwise indicated.

Task	Effects of Susceptibility
Pre-stress	
Continuous access 2-bottle choice	No effect
Light-dark transition test	Reduced entries and time spent in light compartment (females only)
Fear measures	
Generalization test	Increased fear generalization
SEFL test	Increased fear conditioning
Extinction	Impaired fear extinction
Aversive acoustic stimulus	Increased fear conditioning

Anxiety measures	
Open field test	Reduced mobility, blunted response to light onset (males only)
Elevated plus maze	No effect
Light-dark transition test	Reduced entries into light compartment
Alcohol measures	
Intermittent access 2-bottle choice	Increased alcohol consumption and preference (males only)

A key feature of PTSD is exaggerated responses to mild stressors that are reminiscent of the original trauma, which is captured by the SEFL test (Bremner et al., 1995; Dykman et al., 1997; Rau et al., 2005). However, PTSD is also characterized by several other changes in fear behavior. Overgeneralization of fear from the original trauma situation to safe situations is believed to play a key role in PTSD symptomology, and PTSD patients show increased fear generalization from shock-paired cues to safe cues (Kaczkurkin et al., 2017). Here we demonstrate that addition to showing exaggerated fear to both shock and aversive acoustic stimuli, Susceptible subjects show increased generalization from the fear context to a novel context.

Impaired fear suppression is also believed to play an important role in the maintenance of PTSD. Exposure therapy, a common treatment for PTSD, is a form of extinction learning in that subjects are repeatedly exposed to fear or anxiety-producing stimuli in a safe environment (Craske et al., 2008). However, one challenge in treating this disorder is that exposure therapy is often ineffective, and subjects show impaired fear extinction in laboratory settings (Craske et al., 2008; Hembree et al., 2003; Milad et al., 2009). Our results indicate that Susceptible subjects also demonstrate this impairment in fear extinction, though it should be noted that directly comparing extinction rates across Susceptible and Resilient subjects is complicated by the fact that these groups differ in initial freezing levels prior to extinction. To address this issue, we examined the number of extinction sessions needed to suppress freezing to 50% of the initial level of freezing at the start of the extinction session. Using this measure, we still found that susceptible rats took longer to extinguish than rats classified as resilient.

One key aspect of the SEFL procedure is that it can be readily performed in both males and females, while models of stress exposure such as social defeat stress are limited to males (Berton et al., 2006; Golden et al., 2011). Given that women are twice as likely to develop PTSD compared to men even when controlling for trauma type (Kessler et al., 1995, 2017; Breslau et al., 1997), we anticipated that a greater proportion of females would be classified as Susceptible compared to males. However, we found no differences in the rates of susceptibility in males compared to females, suggesting that biological differences alone may be insufficient to explain the disparity in rates of PTSD across genders.

While we found no sex differences in overall rates of stress susceptibility, we did observe sex-specific differences in its behavioral manifestation. During the open field test, both Susceptible and Resilient females retreated from the light which is in agreement with a previous report indicating that footshock exposure does not impact light avoidance on this task in female rats (Godsil et al., 2005). In contrast, Resilient males also showed light avoidance while Susceptible males showed no change in time spent in Zone 4 following light onset.

A major challenge in treating PTSD is the high levels of comorbidity between PTSD and alcohol abuse (Kessler et al., 1995; Mills et al., 2006; Perkonigg et al., 2000). Notably, rates of alcohol abuse are higher in men than in women, as are rates of comorbidity between PTSD and alcohol abuse (Kessler et al., 1995). Mirroring this pattern, we found that stress susceptibility impacted alcohol consumption in a sex-specific manner with susceptibility associated with increased alcohol consumption in males but not in females. Examination of the results indicates that both Susceptible and Resilient females appear to show elevated levels of alcohol intake compared to Resilient males, which corresponds with previous reports that females show elevated alcohol drinking compared to males (Lancaster et al., 1996; Priddy et al., 2017). Lastly, substance use disorders (SUDs) are more likely to follow than precede PTSD (McFarlane, 1998), which was supported by our finding that no differences in alcohol consumption were observed in Susceptible subjects prior to stress exposure.

Identifying the underlying factors that promote stress susceptibility is critical for understanding why some individuals are at risk for developing PTSD. This procedure offers a powerful tool for directly probing the contribution of proposed risk factors to the development of PTSD-related behaviors. Preexisting anxiety disorders, which are also more common in women than in men, have been shown to increase the risk for subsequent PTSD (Breslau et al., 1997; Kessler et al., 2005; McLean et al., 2011). In support of this, we found that pre-stress anxiety levels were predictive of future stress susceptibility in females, but not in males. Increased activation of the hypothalamus-pituitary-adrenal (HPA) axis in females has been hypothesized to underlie the elevated rates of PTSD and anxiety disorders in women and contribute to stress susceptibility (Kudielka and Wüst, 2010; Rao and Androulakis, 2017). It is therefore plausible that increased baseline anxiety in Susceptible females was reflective of uniquely high levels of HPA axis activation in these subjects. Interestingly, a recent study reported that baseline anxiety was not associated with stress susceptibility as assessed by changes in social behavior and fear extinction following stress exposure (Colucci et al., 2020). However, this study employed only male rats, highlighting the need for stress exposure models that can be employed in both males and females.

Within female rodents, estrous cycle has been reported to alter fear conditioning and extinction (Milad et al., 2009b; Cushman et al., 2014). We did not collect estrous cycle data in the present experiments as this would require additional handling of the females for vaginal smear collection that cannot be adequately controlled for in male rats (Hubscher et al., 2005). However, given that baseline anxiety-related behavior was uniquely predictive of stress susceptibility in females, it will be worthwhile to evaluate the potential role of estrous cycle in future studies.

An additional potential limitation of this study is the use of a single classification criterion to determine whether subjects were susceptible or resilient, while PTSD diagnosis includes symptoms not necessarily related to trauma-related memories (APA, 2013). However, one unique strength of our present criterion is that it allowed us to utilize the decades of research using the SEFL model to establish an *a priori* criterion for stress susceptibility, rather than relying on post-hoc classification. Given our findings that Susceptible subjects showed a variety of changes in PTSD-related behaviors, in future studies it will be worth incorporating such measures to identify “PTSD-like” animals, and to investigate whether different groups of subjects show changes in specific types of behaviors, e.g. high-fear animals versus high-anxiety animals.

The SEFL effect lasts a minimum of 3 months and may very well be permanent (Rau et al., 2005; Rau and Fanselow, 2009). An interesting, albeit speculative, question is why does stress produce such long-lasting and pronounced PTSD symptomatology? One possible answer is that it represents a functional adaptation that helps promote survival in a dangerous world. Another is that it is a fully pathological state. Such pathology might occur because while fear is an adaptation to short term emergencies such as predation (Bolles, 1970), prolonged stress pushes this system into overdrive with detrimental results. Some insight into this issue may be provided by the sex differences we observed. Previously, we reported that with the standard 15-shock procedure the overall levels of SEFL were not different between sexes (Poulos et al., 2015). In this current experiment the 4-shock stressor produced no sex differences in the overall level of SEFL or in the proportion of each sex exhibiting the phenotype. However, there were sex differences in the factors that predicted stress susceptibility. These sex differences suggest that while the levels of SEFL susceptibility and magnitude are equivalent between sexes, the mechanisms through which animals reach that level may differ. That the same levels and proportions are obtained via different mechanisms suggests that there is convergent evolution toward these levels. Such convergent evolution is consistent with the hypothesis that PTSD is an adaptation to dangerous environments.

In conclusion, here we demonstrate that a modified version of the stress-enhanced fear learning procedure can be used to produce distinct susceptible and resilient populations that exhibit a constellation of behaviors relevant to PTSD symptomology in both males and females. We show that while some aspects of stress susceptibility are shared across sexes, other aspects including the ability of baseline anxiety to predict future susceptibility manifest differently in males and females. This procedure is a valuable tool for probing the biological mechanisms that support stress resilience.

## Funding

This research was supported by National Institutes of Health R01-AA026530 (MSF, IS) and the Staglin Center for Brain and Behavioral Health (MSF).

## CRediT authorship contribution statement

**Sarah T. Gonzalez:** Investigation, Formal analysis, Writing – original draft. **Vincent Marty:** Investigation, Writing – review & editing. **Igor Spigelman:** Conceptualization, Methodology, Writing – review & editing, Supervision. **Steven P. Reise:** Formal analysis, Writing – review & editing. **Michael S. Fanselow:** Conceptualization, Methodology, Writing – review & editing, Supervision, Funding acquisition.

## Declaration of competing interest

The authors declare that they have no known competing financial interests or personal relationships that could have appeared to influence the work reported in this paper.
