# Supplementary material for: Impact of stress resilience and susceptibility on fear learning, anxiety, and alcohol intake
Source: Neurobiol Stress. 2021 May 6;15:100335. doi: 10.1016/j.ynstr.2021.100335 (PMC8135041; doi:10.1016/j.ynstr.2021.100335)
Supplement: Multimedia component 1 [file mmc1.pdf]

1. Supplementary Methods

1.1. Distribution of SEFL Test scores in males versus females

We investigated whether there were any sex differences in the distribution of SEFL test scores among the unstressed controls or among subjects exposed to the 15-shock stress. The Kolmogorov-Smirnov test was used to compare the distributions of scores of No Stress males (n=137, Mean=18.6%, SD=19.7%) and No Stress females (n=45, Mean=18.9%, SD=19.6%), and to compare the distribution of 15-Shock males (n=98, Mean=75.5%, SD=22.4%) and 15-Shock females (n=32, Mean=71.4%, SD=20.4%). We found no sex differences in either the No Stress condition (D=0.18, p=0.22; Figs S1A-B) or in the 15-footshock condition (D=0.22, p=0.19; Figs S1C-D). We further found that the susceptibility cutoff was the same when calculated separately for females (58%) and males (58%). We performed a similar analysis on the subjects from the current experiment that were exposed to the 4-footshock stress. SEFL Test scores from females (n=22, Mean=54.8, SD=23.6) and males (n=22, Mean=61.9, SD=27.4) are plotted in Figures S1E-F. No difference in these distributions was observed (D=0.23, p=0.63).

1.2. Evaluation of resilience criterion based on subjects exposed 15-shock stress

Our criterion for classifying subjects as “Susceptible” or “Resilient” was based on identifying individuals that showed abnormally high levels of fear compared to that of unstress controls. Given that some individuals experience trauma but do not go on to develop PTSD, an alternative approach would be to determine a cutoff for resilience based on the performance of stress-exposed subjects. To address this possibility, we compiled the SEFL test scores obtained from subjects exposed to the standard 15-footshock stress (n=130). We classified subjects as “Resilient” if they showed performance on the SEFL test that was at least two standard deviations below the means of subjects exposed to the standard 15-footshock stress, which based on this dataset was 30.5% freezing.

We found that this alternative approach did not perform as well for several reasons. First, while only a small proportion of people who experience trauma go on to develop PTSD, this criterion would result in 86.4% of subjects exposed to the 4-footshock stress being classified as "Susceptible". Second, the 58% criterion corresponds to the separation within the bimodal distribution that was observed in the 4-footshock subjects, while the 30.5% criterion does not (Figure 1C). Thus, we determined that the 58% criterion based on the unstressed controls was most appropriate.

1.3. Fear, anxiety and alcohol intake in Resilient and Susceptible subjects compared to No Stress subjects

To illustrate how performance of Susceptible and Resilient animals compared to that of unstress controls on the battery of fear, anxiety and alcohol intake assessments used in this experiment, 62 adult male and female Long-Evans rats (Envigo) concurrently received identical treatment to as those described in Methods, but on Day 16 of the procedure received 90 minutes of context exposure without footshock. 42 subjects received alcohol prior to stress, while 20 subjects did not. Means and standard deviations for each task for Resilient, Susceptible and No Stress groups are shown in Table S1. For tasks involving repeated measures, the average value across all time points was computed.

1.4. Enhancement of fear to a novel aversive stimulus following extinction of fear generalization

1.4.1. Animals

To assess whether susceptible subjects showed enhanced fear to a novel aversive stimulus, 45 adult male and female Long-Evans rats (Envigo) approximately 79 days old at the start of the experiment were used. Animals were individually housed under a 12-hour light/dark cycle. Food and water were available ad lib in the home cage. Animals were handled daily for 60

seconds each day for 1 week prior to the start of the experiment. The Chancellor's Animal Research Committee at UCLA approved all procedures involving animals.

1.4.2. Stress-enhanced fear learning

Training took place in two sets of four identical fear conditioning chambers housed in sound-attenuating shells (Med-Associates). Context A (trauma context) contained flat grid floors, was lit by a white house light and scented with 50% Windex solution. Ventilation fans provided background noise. Context B (mild stressor context) contained a black triangular insert and floors composed of vertically alternating grid bars, was lit by a near-infrared light and scented with 1% acetic acid. Each set of grids was wired to a shock generator and scrambler. Stimulus delivery was controlled and freezing automatically scored using VideoFreeze software (Med Associates Inc, VT).

Subjects were first transported to Context A in their homecages where they received a traumatic stressor consisting of 4 1-sec, 1-mA unsignalled footshocks. Fear to the trauma context was assessed by returning subjects to Context A the following day for 8 minutes without footshock. The next day subjects were transported to Context B in a black plastic tub divided into 4 quadrants. Following a 3-minute baseline period, all subjects received a mild stressor consisting of a single 1-sec, 1-mA footshock and were removed 30 seconds later. All subjects were returned to Context B the following day for 8 minutes to assess fear to the mild stressor context.

1.4.3. Aversive acoustic stimulus

Training took place in a third set of four identical fear conditioning chambers. Context C contained a curved white plastic wall and white plastic floor inserts. The apparatus was lit by near-infrared light and scented with 1:30 Simple Green solution. Acoustic stimuli were delivered using Goldwood GT-1005 wide dispersion piezo tweeters mounted to the wall of the chambers and connected to an amplifier.

Subjects first underwent 4 days of extinction training to reduce differences in baseline fear to Context C. Subjects were exposed to Context C for 30 minutes per day without footshock delivery. Training occurred the day after completion of fear extinction. Following a three-minute baseline period, all subjects received a 100-msec, 110-dB burst of white noise and were removed 30 seconds later. One day later subjects were returned to Context D for 8 minutes to assess fear to the context.

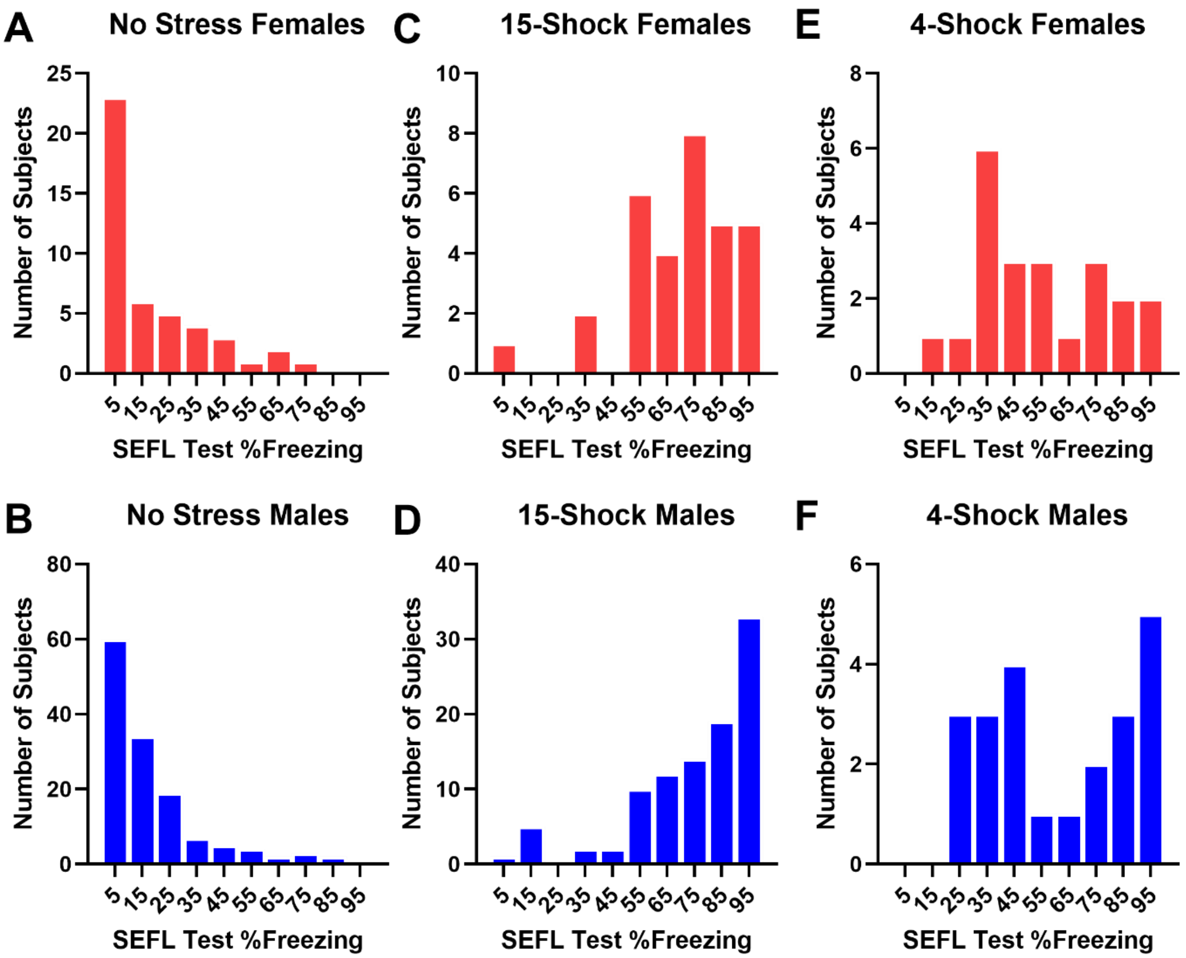

Figure S1. Distribution of SEFL Test scores in males versus females following different levels of stress. **A-B.** Distribution of SEFL test scores in **(A)** unstressed females (n=45) and **(B)** unstressed males (n=137). No differences were observed in the distribution of scores. **C-D.** Distribution of SEFL test scores in **(C)** females exposed to standard 15-footshock stress (n=32) and **(D)** males exposed to standard 15-footshock stress (n=98). No differences were observed in the distribution of scores. **E-F.** Distribution of SEFL test scores in **(E)** females exposed to 4-footshock stress (n=22) and **(F)** males exposed to 4-footshock stress (n=22). No differences were observed in the distribution of scores. Tick marks indicate center of 10% bins.

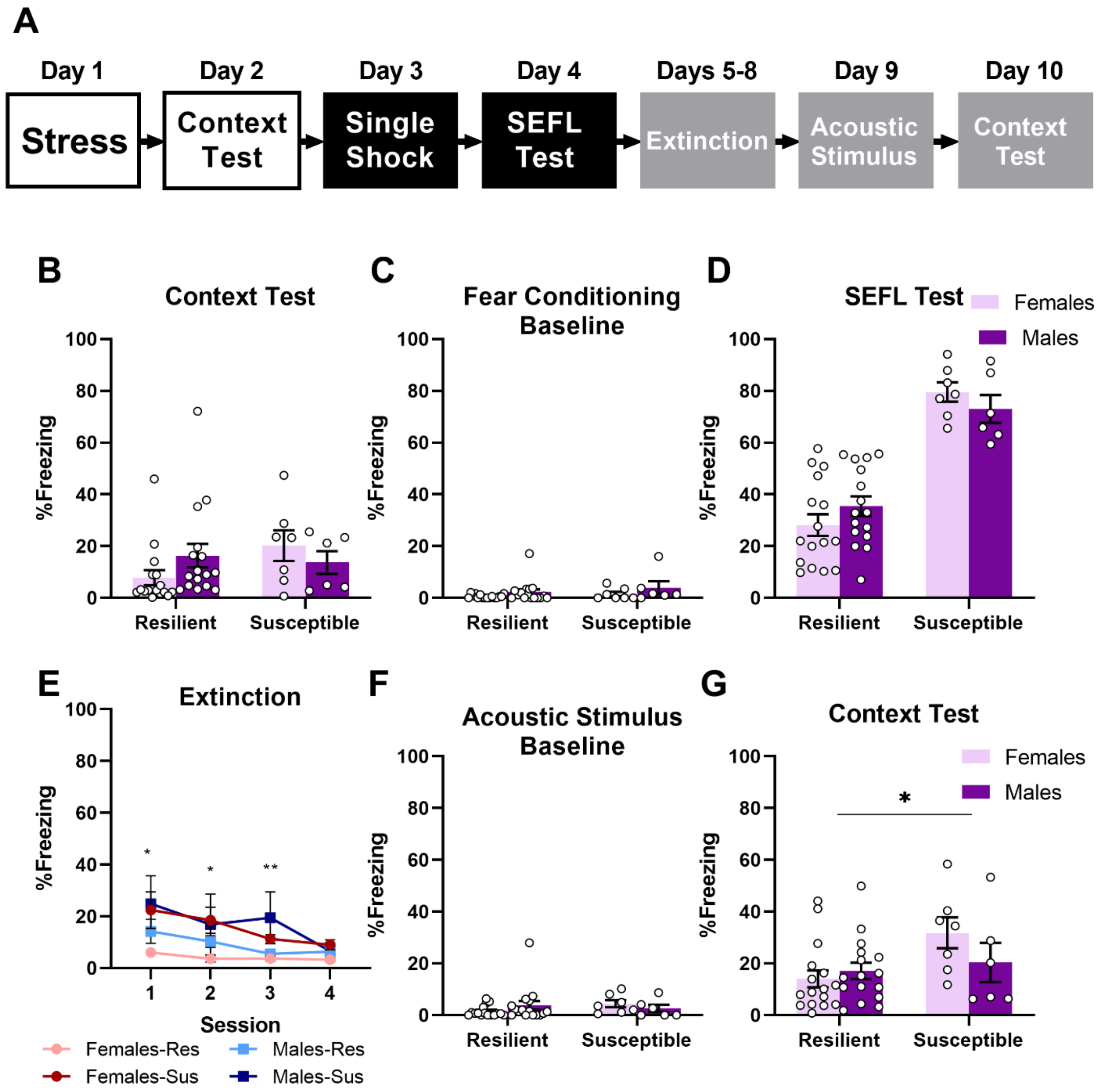

Figure S2. Susceptible subjects show increased fear to a context associated with a brief loud noise following extinction of fear generalization. **A.** Experiment timeline. **B.** Susceptible and Resilient subjects do not differ in fear to stress context (Day 2; $F_{1,41}$=0.99, p=0.33). Freezing scores were low, possibly due to within-session extinction. **C.** No differences in fear during the 3 minutes prior to footshock delivery (Day 3; $F_{1,41}$=1.38, p=0.25). **D.** No sex differences during SEFL Test (Day 4; $F_{1,41}$=0.01, p=0.94). **E.** Susceptible subjects initially show increased fear to acoustic stimulus context but extinguish to the level of Resilient subjects (Days 5-8; $F_{3,123}$=3.13, p=0.03). Data points show freezing during the first 5 minutes of each session. **F.** No differences in fear to the acoustic stimulus context during the 3 minutes immediately prior to stimulus delivery (Day 9; $F_{1,41}$=0.31, p=0.58). **G.** Susceptible subjects show increased fear to acoustic stimulus context (Day 10; $F_{1,41}$=5.12, p=0.03). *p<0.05, **p<0.01. Error bars represent standard error of the mean.

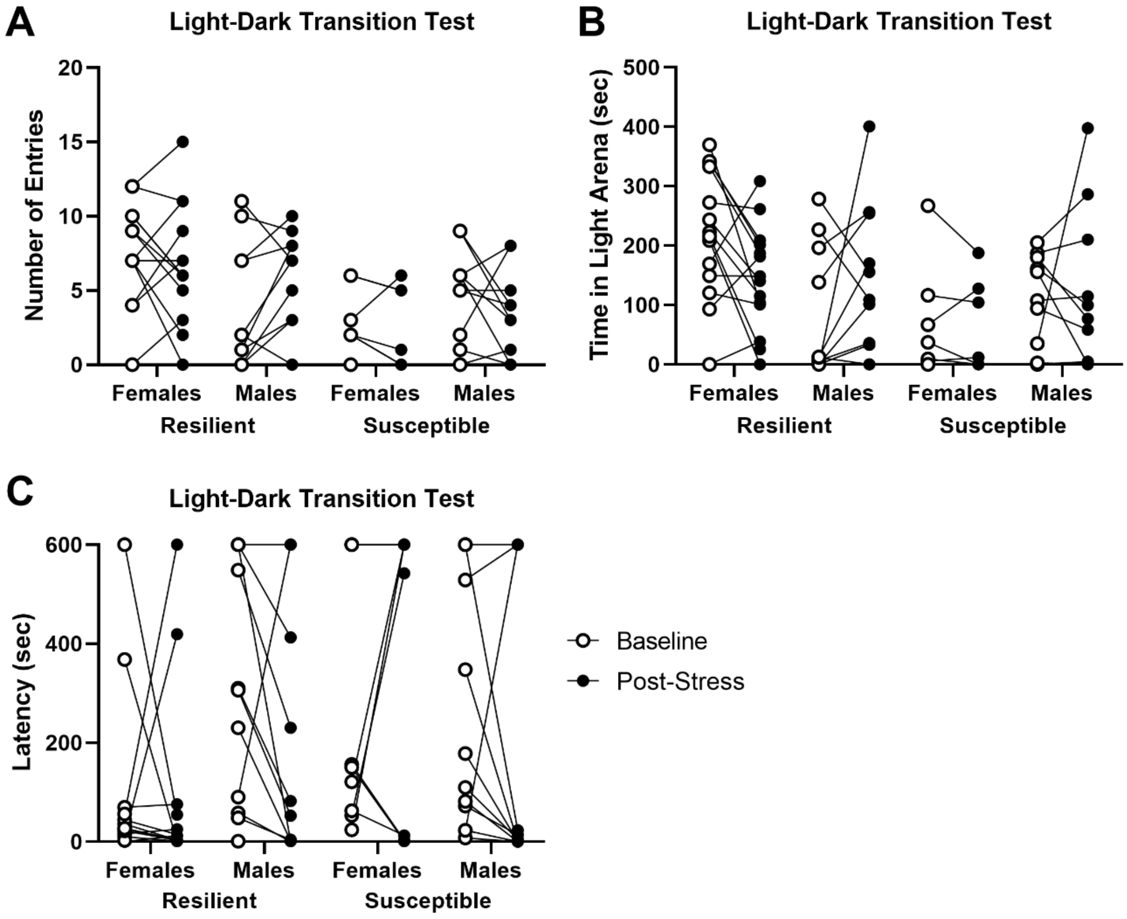

Figure S3. Performance on light-dark transition test prior to and following stress. No interactions between stress exposure and resilience were observed on **(A)** number of entries into the light compartment, **(B)** time spent in the light arena or **(C)** latency to enter the light arena. Each line represents an individual subject.

Task	*Resilient Mean(SD)*	*Susceptible Mean(SD)*	*No Stress Mean(SD)*
Pre-stress			
CA 2-BC (EtOH consumption, mg/kg)	1.7(1.0)	1.8(1.7)	1.7(1.2)
CA 2-BC (EtOH preference)	20.8(12.2)	13.1(13.4)	16.5(10.9)
Light-dark transition test (entries)			
Females	7.5(3.3)	2.6(2.3)	6.5(3.2)
Males	3.7(4.2)	4.5(3.3)	4(3.5)
Light-dark transition test (light time, sec)			
Females	220(107.3)	62.8(92.1)	178.8(108.8)
Males	79.3(108.7)	102.7(81.1)	94.1(88.4)
Light-dark transition test (latency, sec)			
Females	92.1(173.3)	221(238.5)	143.4(179.2)
Males	308.4(243.2)	233.6(240.2)	293.5(241)
Fear measures			
Generalization test (%freezing)	5.7(6.9)	27.4(25.2)	1.9(1.4)
SEFL Test (%freezing)	38.5(10.9)	84.4(11.5)	18.2(15.6)
Extinction (final session %freezing)	7.6(8.8)	29.9(28.4)	5.3(7.3)
Extinction (trials to 50%)	2.1(1.1)	3.4(1.4)	2.5(1.7)
Acoustic stimulus test (%freezing)	19.2(15.8)	49.1(21.6)	7.8(8.9)
Anxiety measures			
Open field test (velocity, cm/sec)	7.5(1.8)	5.2(2.3)	7.3(1.8)
Elevated plus maze (open arms time, sec)	62.9(42.7)	43(24.9)	47.8(35.1)
Light-dark transition test (entries)	6.2(3.7)	2.8(2.8)	6(4.4)
Light-dark transition test (light time, sec)	141.6(104.4)	88.4(113.2)	136.1(112.2)
Light-dark transition test (latency, sec)	128.1(213)	252.8(298.2)	176(243.4)
Alcohol intake			
IA 2-BC (EtOH consumption, mg/kg)			
Females	4.3(1)	4.1(0.9)	4.3(0.8)
Males	2.8(1.3)	4.5(1.3)	4.2(1.5)
IA 2-BC (EtOH preference)			
Females	61.6(12.6)	55.7(15)	61.4(13.4)
Males	47.2(19.4)	75.4(11.8)	73.3(24.8)

Table S1. Comparison of Susceptible and Resilient subjects with No Stress subjects run through the same procedures. Intermittent access 2-bottle choice (IA 2-BC) measures are from subjects that did not receive alcohol prior to stress. All other measures are from subjects that did receive alcohol prior to stress.
